# Effects of Build Orientation on Surface Morphology and Bone Cell Activity of Additively Manufactured Ti6Al4V Specimens

**DOI:** 10.3390/ma11060915

**Published:** 2018-05-29

**Authors:** Volker Weißmann, Philipp Drescher, Hermann Seitz, Harald Hansmann, Rainer Bader, Anika Seyfarth, Annett Klinder, Anika Jonitz-Heincke

**Affiliations:** 1 Faculty of Engineering, University of Applied Science, Technology, Business and Design, Philipp-Müller-Str. 14, 23966 Wismar, Germany; 2 Biomechanics and Implant Technology Research Laboratory, Department of Orthopedics, Rostock University Medical Centre, Doberaner Strasse 142, Rostock 18057, Germany; rainer.bader@med.uni-rostock.de (R.B.); anika.seyfarth@uni-rostock.de (A.S.); annett.klinder@med.uni-rostock.de (A.K.); anika.jonitz-heincke@med.uni-rostock.de (A.J.-H.); 3 Fluid Technology and Microfluidics, Faculty of Mechanical Engineering and Marine Technology, University of Rostock, 18059 Rostock, Germany; philipp.drescher@uni-rostock.de (P.D.); hermann.seitz@uni-rostock.de (H.S.); 4 Institute for Polymer Technologies e.V., Alter Holzhafen 19, 23966 Wismar, Germany; h.hansmann@ipt-wismar.de

**Keywords:** Ti6Al4V, biomaterial, osteoblast, collagen, selective laser sintering, electron beam melting

## Abstract

Additive manufacturing of lightweight or functional structures by selective laser beam (SLM) or electron beam melting (EBM) is widespread, especially in the field of medical applications. SLM and EBM processes were applied to prepare Ti6Al4V test specimens with different surface orientations (0°, 45° and 90°). Roughness measurements of the surfaces were conducted and cell behavior on these surfaces was analyzed. Hence, human osteoblasts were seeded on test specimens to determine cell viability (metabolic activity, live-dead staining) and gene expression of collagen type 1 (Col1A1), matrix metalloprotease (MMP) 1 and its natural inhibitor, TIMP1, after 3 and 7 days. The surface orientation of specimens during the manufacturing process significantly influenced the roughness. Surface roughness showed significant impact on cellular viability, whereas differences between the time points day 3 and 7 were not found. Collagen type 1 mRNA synthesis rates in human osteoblasts were enhanced with increasing roughness. Both manufacturing techniques further influenced the induction of bone formation process in the cell culture. Moreover, the relationship between osteoblastic collagen type 1 mRNA synthesis rates and specimen orientation during the building process could be characterized by functional formulas. These findings are useful in the designing of biomedical applications and medical devices.

## 1. Introduction

The use of metallic materials in clinical applications, e.g., for orthopedic implants, is state-of-the-art. Titanium and its alloys are often used [1]. It is known that additively manufactured scaffolds or implants for biomedical applications can meet both mechanical and biological requirements [2]. The use of additive manufacturing implants in biomedical applications opens up an interesting research field. This topic is well researched in the area of dental or orthopedics applications and also in the field of maxillofacial reconstructions [3,4,5,6,7,8].

In particular, the additive manufacturing processes (electron beam melting—EBM or selective laser melting—SLM) and their parameter variations as well as geometrical variations [9,10] influence the properties of materials [11,12].

In the evaluation of components and their properties, the mechanical and structural properties and their relationships are important. This is especially relevant for open, load-bearing structures [13,14,15,16]. The basic geometric shapes and characteristic sizes of open, load-bearing structures also influence the behavior of human osteoblasts [17,18,19,20].

The surface properties of an additively fabricated porous structure (such as when used on an implant) determine its interaction with the biological environment. It is essential that the topography and chemical conditions on the surface can influence cell adhesion and proliferation [21]. Surface orientation during the building process also has effects on surface roughness and the resulting shape [22]. Pyka et al. [23] reported that this is influenced by processing conditions during additive manufacturing and post-treatment of the surfaces, in particular, for open-porous Ti6Al4V structures. As a result, porous structures with tailored surface roughness can be fabricated. The influence of surface roughness on functional properties can thus be described in relation to the cause.

Important for successful ingrowth of the implant into the surrounding bone is the biocompatibility of the material. Open-porous titanium (or titanium alloys) implants have shown their capability for successful osteointegration. Fassina et al. [24] evaluated the proliferation of human osteosarcoma cells (SAOS-2) on a sintered titanium grid. They described the influence of an electromagnetic stimulus on cell proliferation The focus was on the assessment of cell colonization of coated surfaces of a titanium grid and interaction with the bone matrix [24]. Hollander et al. [25] assessed the amount of proliferation, together with the differentiation of cells on porous Ti6Al4V specimens with varying pore diameters and non-porous and porous blasted surfaces. Ponader et al. [26] investigated the suitability of different EBM-fabricated scaffolds as carriers for bone formation in an in vivo study. Here, smooth and porous Ti6Al4V structures were investigated. Cox et al. [4] evaluated the influence of the finishing surface on the topography of implants. The selectively laser-melted (SLM) parts showed significant changes related to cellular activities [4]. Wang et al. [27] investigated the biocompatibility of Ti6Al4V implants (manufactured by SLM and EBM) in vitro and in vivo. Among other parameters, they assessed the biocompatibility and cytocompatibility and showed no significant differences between titanium specimens manufactured by EBM and SLM [27].

In addition to the roughness of surfaces, the structure of pores also influences cell growth. This concerns the pore size and shape as well as the cell interconnectivity [19,28,29]. Lincks et al. [30] found, that for example, a rough microtopography on a pure titanium surface offers optimal conditions for orthopedic implants.

Important for optimal cell growth are the geometric conditions of open-pored structures. The optimal conditions are pore sizes between 50 to 500 µm and a porosity of 60–75% [31,32]. The geometric conditions directly affect the migration of cells, the transport of nutrients, the matrix deposition and the vascularization [28].

The study of Alvarez et al. [28] aimed to describe differences in cell viability and bone formation as a function of surface quality. In contrast to other studies, we specifically analyzed the influence of orientation in the production process on surface design in the study presented. Existing studies have dealt with the influence of surface roughness and cell growth in combination with pore size as well as the porosity of the considered scaffolds.

In our present study, the influence of roughness, influenced by the orientation in the building process, was considered without considering the property-influencing factors, such as pore size and porosity, into account. For this purpose, titanium constructs with three geometrical orientations (0°, 45° and 90°) were fabricated in SLM and EBM manufacturing processes. Furthermore, the viability and gene expression rates of osteoblastic differentiation markers were analyzed with respect to the surface roughness of the test specimens.

## 2. Materials and Methods

### 2.1. Design and Fabrication of Specimens

CAD design software (PTC Creo, Version 2.0, Parametric Technology Corporation, Needham, MA, USA) was used to design the specimens. The data sets of the CAD samples were used to prepare the test specimens. The SLM parts were manufactured by C.F.K CNC-Fertigungstechnik Kriftel GmbH (Kriftel, Germany) on an SLM 280 using SLM technology.

The EBM parts were produced by the Faculty of Mechanical and Marine Engineering of the University of Rostock using an electron beam melting system A1. For both manufacturing processes the parameters, the pre- and post-treatment of the parts as well as the used materials correspond to the description in [33].

**Figure 1:**
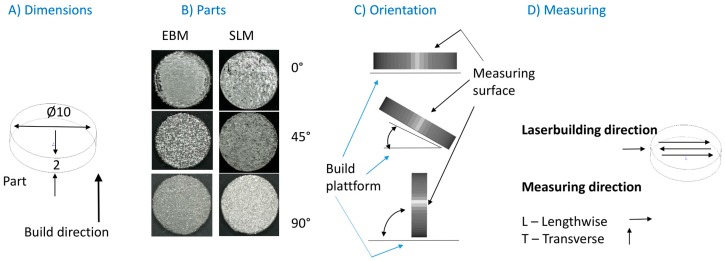
Design of the Ti6Al4V test specimens. (**A**) Specimens with dimensions (mm) as shown were built in three build orientations (**B**,**C**) using two manufacturing systems; surface measurement (Ra) was carried out in two directions (lengthwise and transverse) on the part surface (**D**).

The parts were aligned and manufactured in such a way that the surface to be examined was not influenced by the treatment (pre- or post-treatment). The supports (typical for this kind of manufacturing process) were located at the bottom side of the 0° and 45° oriented samples. For the 90° oriented samples, the supports were on the narrow side of the geometry. Both surfaces were used for the evaluation (see Figure 1C).

### 2.2. Measurements

Surface quality measurements were performed on five specimens using a 3D digital laser-scanning microscope VK-X260 (KEYENCE Deutschland GmbH, Neu-Isenburg, Germany) compliant with ISO 25178-2 [34]. The roughness value (Ra) was determined as the arithmetic average of the absolute values for the manufactured constructs in all build orientations. Measurements were elaborated (see Figure 1) lengthwise (in direction of the laser course) and transversely (90° to the laser course). The measuring lines (*n* = 9) were carried out over a distance of 4 mm with a cut-off wavelength (λ_C_) of 0.25 mm. The distance between the lines was 40 μm. The average arithmetic height (Sa) was determined as the arithmetic mean height of the surface for the SLM and EBM constructs in all build orientations. An area of 1000 × 1000 μm on every part was measured (see Figure 1), and the λ_C_ used was 0.25 mm. To avoid the influence of possible support residues, measurement was carried out on the support structure-free surfaces of the parts (see Figure 1). For the 90° oriented specimens, the measurement was made on a randomly selected side.

### 2.3. Cell Biological Tests

Human primary osteoblasts (three male donors: mean age 63.0 ± 17.8 years) were isolated under sterile conditions from bone marrow derived from the femoral heads of patients undergoing primary hip replacements. All samples were collected after participants had signed written informed consent forms. The study was conducted in accordance with the Declaration of Helsinki and was approved by the Local Ethics Committee of University of Rostock (AZ no.: 2010-10). Cell isolation and cultivation was done according to the protocol of Jonitz-Heincke et al. [2]. Sterile test specimens were placed in standard 24-well cell culture plates (in duplicates), and aliquots of 30,000 cells/specimen in 200 µL cell culture medium droplets were seeded on top of the specimens allowing cell adherence for 30 min. Afterwards, the cell-seeded specimens were carefully overlaid with complete cell culture medium, DMEM (Dulbecco’s modified Eagle medium), supplemented with 10% fetal calf serum (both: PAN Biotech, Aidenbach, Germany), 1% hepes buffer, 1% penicillin/streptomycin (both: Invitrogen, Darmstadt, Germany), and 1% amphotericin B (Biochrom AG, Berlin, Germany) containing osteogenic additives (50 μg/mL l-ascorbate-2-phosphate, 10 mM β-glycerophosphate, 100 nM dexamethasone (all: Sigma-Aldrich, Munich, Germany)). Cells were incubated under standard culture conditions at 37 °C and 5% CO_2_. After 3 and 7 days of incubation, cell viability was analyzed by the water soluble tetrazolium salt (WST-) 1 assay (Roche, Penzberg, Germany) which indicates metabolic activity as well as live-dead staining (Invitrogen). This process was performed according to the manufacturer’s recommendations.

Therefore, the medium was removed from the osteoblasts, replaced by a defined volume of WST-1/DMEM (ratio 1:10) and incubated for 60 min at 37 °C and 5% CO_2_. After the incubation period, supernatants were collected and transferred into a 96-well cell culture plate (in duplicate) to determine the absorption at 450 nm (reference wave length: 630 nm) in a microplate reader (Dynex Technologies, Denkendorf, Germany). The adherent cells were washed with phosphate buffered saline (PBS) and subsequently incubated with a defined volume of live-dead dye (Invitrogen) in PBS (Calcein AM:ethidium homodimer 1:PBS; 1:100:2000) at room temperature in the dark. Images of live (green fluorescence, Calcein AM) and dead cells (red fluorescence, ethidium homodimer 1) were taken under a fluorescence microscope (Nikon ECLIPSE TS100, Nikon GmbH, Duesseldorf, Germany) at the same position. Afterwards, pictures of living and dead cells were overlaid using the image processing software, Adobe Photoshop CS6 (Adobe Systems Software Ireland Ltd., Dublin, Ireland).

Additionally, total RNA (ribonucleic acid) was extracted by lysing the cells directly from the top of the specimen using the peqGOLD Total RNA kit (PeqLab Biotechnology GmbH, Erlangen, Germany) according to the manufacturers’ recommendations. RNA was eluted in 25 µL diethyl pyrocarbonate (DEPC) water, and its concentration was measured using the Tecan-Reader Infinite^®^ 200 Pro (Tecan Trading AG, Mannedorf, Switzerland) with RNase free water as the blank. The mean ratio of isolated RNA (absorbance at 260/280) was 2.0. An amount of 100 ng of isolated RNA from each sample was used for cDNA synthesis (High Capacity cDNA kit; Applied Biosystems, Foster City, CA, USA) and processed according to the manufacturer’s instructions. Relative quantification of target cDNA levels was performed by quantitative real-time PCR (polymerase chain reaction (qTower 2.0; Analytik Jena AG, Jena, Germany)) using the InnuMIX qPCR MasterMix SyGreen (Analytik Jena AG) and cDNA primers (Col1A1: acgaagacatcccaccaatc (fwd), agatcacgtcatcgcacaac (rev); MMP1 (matrix metalloproteinase-1): gagcagatgtggaccatgc (fwd), tcccgatgatctcccctgac (rev); TIMP1 (metallopeptidase inhibitor 1): attgctggaaaactgcaggatg (fwd), gtccacaagcaatgagtgcc (rev); HPRT (housekeeper): ccctggcgtcgtgattagtg (fwd), tcgagcaagacgttcagtcc (rev); all: Sigma Aldrich). Quantitative real-time PCR was performed according to the following process: 95 °C for 2 min, 40 cycles at 95 °C for 5 s and 65 °C for 25 s. The reactions were performed in duplicates. The relative expression of each mRNA compared with HPRT was calculated by the equation ΔCt = Ct_target_ − Ct_HPRT_, and the relative amount of target mRNA in cells was expressed as 2^−(ΔCt)^.

### 2.4. Statistical Analysis

All results listed in tables are mean values ± standard deviations (SD). The interrelationship among the means of Sa and Col1A1 as well as the relationship between the bone degrading factors, MMP 1 and TIMP 1, were assessed by linear regression using Excel 2016 for Windows. All statistical analyses were conducted using GraphPad Prism 7.02 (GraphPad Software, La Jolla, CA, USA). The relationship between the roughness Sa (means) and the orientation was statistically evaluated with the Kruskal–Wallis analysis. As a post-hoc test, the Dunn’s test was performed. The results from this comparison were diagrammed in a boxplot.

For evaluation of the metabolic activity and expression of the specific bone differentiation marker, three independent experiments were carried out by testing primary osteoblasts from three different donors on the two differently manufactured samples at two time points and three different surface orientations. Therefore, a repeated measure two-way ANOVA was performed using the two manufacturing methods at both time points and the different surface orientations as variables. Additionally, a Bonferroni’s post-hoc test was performed to statistically examine significant differences between the means. Post-hoc tests were performed within a manufacturing method for both time points comparing the different surface orientations as well as the different manufacturing methods and time points at the same surface orientation, respectively. Bonferroni’s correction for multiple comparisons was used in statistical hypothesis testing as three comparisons per family were carried out. A significance level of *p* < 0.05 was considered to be statistically significant.

## 3. Results 

### 3.1. Characteristics of and Roughness of Part Surfaces 

**Figure 2:**
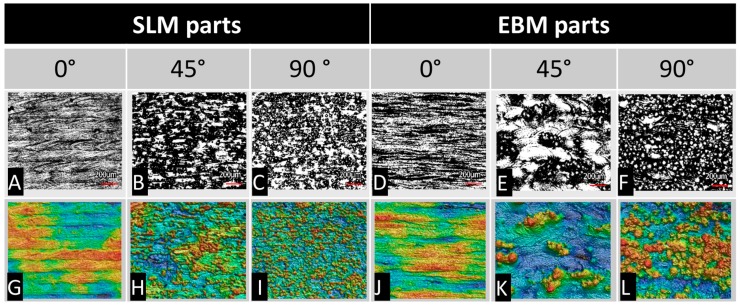
Overview of the surface characteristics of the measured surfaces on manufactured specimens in relation to the build orientation (0°, 45° and 90°) and the manufacturing process (selective laser melting, SLM; electron beam melting, EBM). The pictures in the first row (**A**–**F**) represent the optical surfaces (bar: 200 µm) which are displayed as false-color images in the row below (**G**–**L**). The highest position is displayed in red, while the lowest position is displayed in blue.

**Figure 3:**
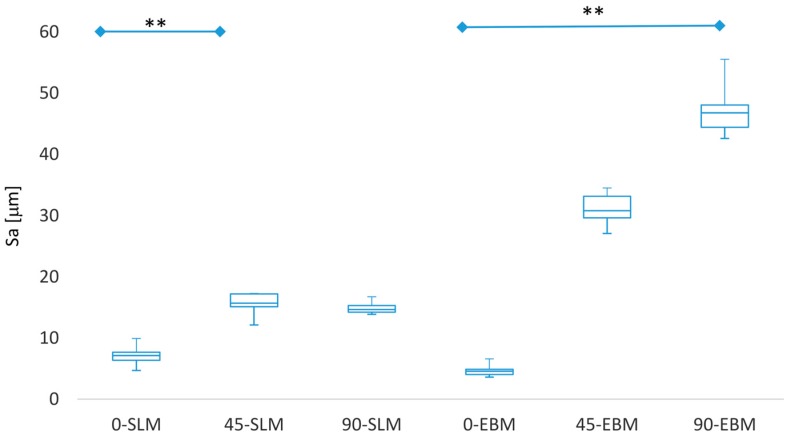
Measured Sa values for the specimens (SLM, EBM) illustrated with a box plot. The figure indicates the median values, the interquartile range and the outlier values (*n* = 5). The IQR represents the interval between the 25th and 75th percentile with a blue box. The bars at the top of the figure indicate the statistical significance differences (significance level ** *p* < 0.01; Kruskal–Wallis test with Dunn’s post-hoc test) between the values.

The test specimens were analyzed with a 3D digital laser-scanning microscope to characterize the surfaces produced by SLM and EBM (Figure 2). To evaluate the surface quality of the parts, a roughness measurement was performed (Table 1). The results from the Sa measurements are shown in Figure 3.

The 3D digital laser-scanning microscope images show the superficial differences arising as a result of different orientations during the manufacturing process. The 0° oriented samples (A, D → G, J) show very smooth surfaces characterized by the presence of linear structures. The linear structures represent the scan paths of the laser during the manufacturing process. In the corresponding false color images (G, J), minor height differences become apparent. The 45° oriented (B, E → H, K) and 90° oriented samples (C, F → I, L) show clear signs of the melted adherent powder particles on the surface. The height differences visible in the false-color images were slightly more pronounced in the 90° oriented SLM sample (I) than in the EBM sample (L).

The Ra values received for the manufactured Ti6Al4V specimens corresponded with trends in the literature. Triantaphyllou et al. [35] identified Ra values for SLM parts between 8 and 14 μm (0 and 90° orientation). The Ra values for EBM parts differed between 8 to 30 μm (0 and 90° orientation) [35]. A number of factors influence the surface roughness. These include the orientation during the building process, the laser beam diameter and the powder (size distribution, layer thickness). The value differences can also be caused by manufacturing parameters. Suard et al. [22] confirmed the tendency of increasing surface roughness from additive manufactured parts from vertical to tilted specimens. Tuomi et al. [36] determined Ra values of 30 μm for 0° oriented EBM samples.

In both methods, and in both measuring directions, the Ra values for 0° were the smallest. Both methods produced less roughness in the lengthwise direction (direction of laser track). The SLM constructs showed lower values in comparison to the EBM constructs. The differences between the 45° and 90° oriented samples were smaller for the SLM constructs in both measuring directions than those for EBM. The roughness differed significantly between 0° and 90° for EBM specimens (*p* = 0.0012) and between 0° and 45° for SLM specimens (*p* = 0.0089).

### 3.2. Cell Viability and Surface Roughness

**Figure 4:**
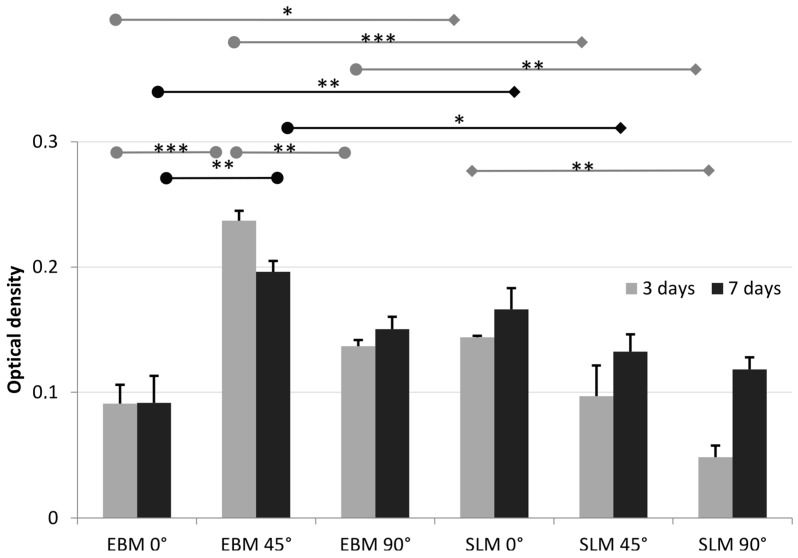
Metabolic activity of human osteoblasts seeded on the EBM and SLM specimens (*n* = 3) after three and seven days of cultivation. Results are shown as mean values including standard deviations. The bars at the top of the figure indicate significant differences (significance level * *p* < 0.05; ** *p* < 0.01; *** *p* < 0.001, repeated measures two-way ANOVA with Bonferroni’s post-hoc test) between the mean values.

**Figure 5:**
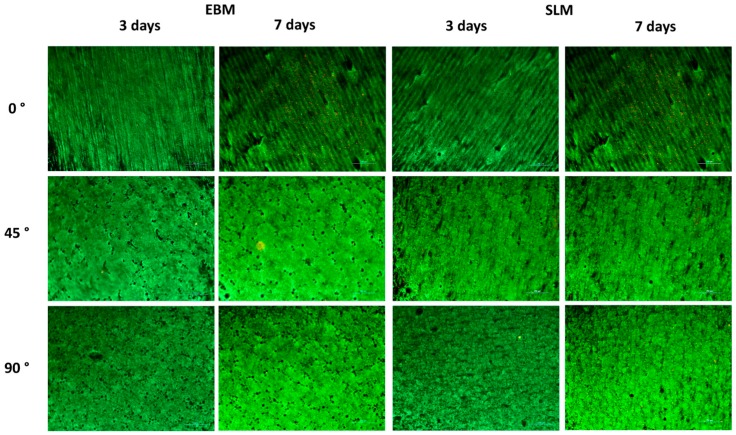
Live-dead staining of human osteoblasts on the EBM and SLM titanium specimens in the three different build orientations on day 3 and day 7 (*n* = 3; green = living cells; red = dead cells; scale bar: 250 μm).

The viability of human osteoblasts was studied by seeding cells on titanium specimens made by EBM and SLM (Figure 4). The results of the live-dead staining from human osteoblasts are shown in Figure 5.

The metabolically active human osteoblasts were determined on the surfaces of the different test specimens (Figure 4). For the EBM constructs, a significant increase in metabolism occurred between 0° and 45° (*p* = 0.0003 (3 days); *p* = 0.0032 (7 days)). Between 45° and 90°, however, there was a significant decrease in metabolic activity (*p* = 0.0041 (3 days)). For the SLM constructs, a slight reduction in cellular metabolism was shown with increasing surface orientation. A statistically different value was only shown between 0° and 90° after 3 days of incubation (*p* = 0.0049). Comparing the EBM and SLM specimens, significant differences were true for all orientations after 3 days (*p* = 0.0393 (0°); *p* < 0.0001 (45°) *p* = 0.0012 (90°)) as well as after 7 days (*p* = 0.009 (0°); *p* = 0.0242 (45°)).

For all test specimens, a good cell distribution with a confluent cell layer was shown. The orientation of cell growth correlated with the surface orientations, as shown in Figure 2. Similar to our results for cellular metabolism, more cells seemed to grow on the 45° orientated EBM surface. A higher number of dead cells was visible for both EBM 0° (7 days) and SLM 0° (7 days). However, differences in the brightness of green-fluorescent cells resulted from the surface orientation which influenced the fluorescence signal.

### 3.3. Roughness and Induction of Bone Formation and Degradation

**Figure 6:**
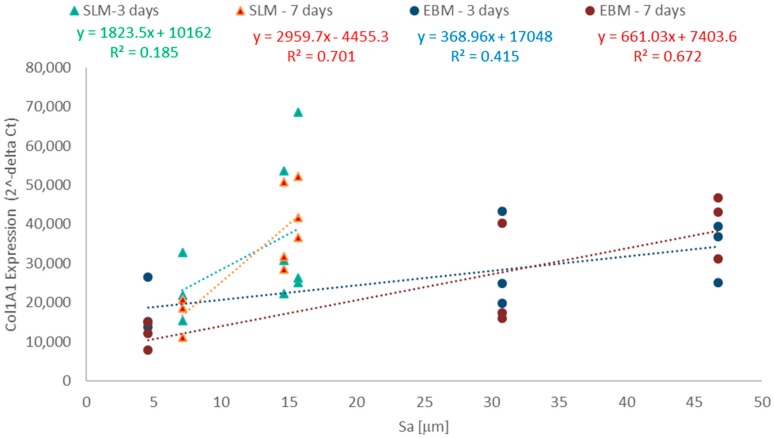
Correlation between the surface roughness (Sa) and collagen synthesis of human osteoblasts. The osteoblasts were cultured under static conditions (3 and 7 days).

**Figure 7:**
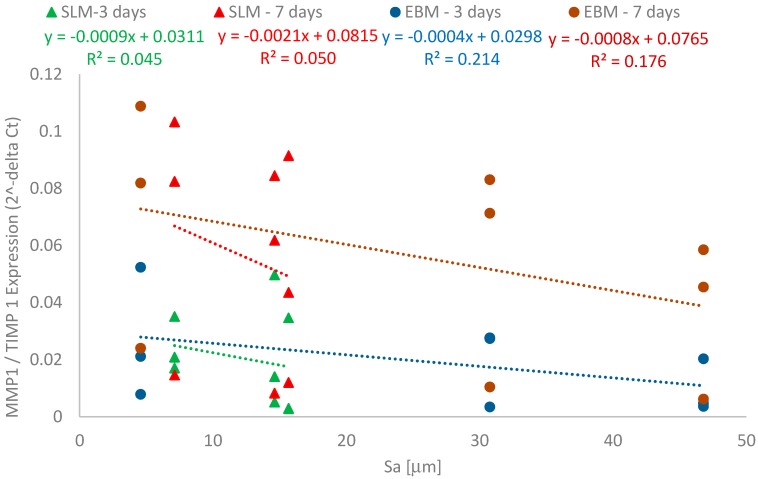
Correlation between the surface roughness (Sa) and the bone degradation factors, *MMP 1* and *TIMP 1*.

The influence of both the fabrication processes and the three surface orientations on the induction of bone formation and degradation was characterized by gene expression analyses. Table 2 shows the gene expression results of the specific bone differentiation and formation marker, *Col1A1*, and the bone degrading marker, *MMP1*, and its natural inhibitor, *TIMP1*, in response to the manufactured SLM and EBM constructs. The correlation between bone formation and roughness is shown in Figure 6. The relationship between *MMP1* to *TIMP1* proportions and roughness is shown in Figure 7. Since all test specimens were manufactured additively, we used the equation method of 2^−ΔCt^ in order to determine differences dependent on surface orientation.

The representative data in Figure 6 and Figure 7 were specifically chosen, and in each case, all three values measured in the biological tests are shown plotted against the mean values for the determined roughness (Sa). The low variability in roughness permits this form of representation which improves the clarity and, at the same time, the trustworthiness of the linear correlation.

The surface orientations obtained with both production variations strongly influenced the *Col1A1* synthesis rates of human osteoblasts. Comparing the results, it was noticeable that cells on the 0° oriented samples had a lower mRNA expression levels compared to the 45° and 90° oriented samples. A significantly higher expression rate on the SLM specimens with 45° orientations was detected after 7 days compared to those with 0° orientations (*p* = 0.0322). The same was true with EBM for the 90° orientations compared to the 0° orientations (*p* = 0.0023). Interestingly, similar expression levels were determined for both SLM 45° and EBM 90° after 7 days. For all test specimens, no time-dependent differences were notable.

The induction of the bone degradation marker *MMP1* was very weak while induction of *TIMP1* expression was very strong, with no differences shown between manufacturing processes and surface orientations. The *MMP1/TIMP1* ratio was smaller than 1, indicating that there was no induction of bone degradation processes (see Figure 7). Our results therefore indicate that the surfaces promote bone formation processes while degradation is not induced.

Both manufacturing processes showed an identical influence on bone formation processes. Collagen type 1 synthesis rates of human osteoblasts were enhanced with increasing roughness (Sa). Cells on SLM constructs showed higher collagen synthesis rates compared to the EBM constructs.

A linear relationship was found between *Col1A1* synthesis rates and roughness (Sa) for both manufacturing processes. SLM constructs showed a higher correlation than the EBM parts after 7 days. The presented functional relationship regarding the collagen synthesis was confirmed by statistical methods. Here, the two-way ANOVA with Bonferroni’s post-hoc test demonstrated a significant influence between the mean values of bone formation processes. After 7 days, a significance level was determined for the EBM components and the SLM components of less than 0.01 (EBM 0-90° *p* = 0.0023) and less than 0.05 (SLM 0-45° *p* = 0.0322), respectively. This proves statistically that there is a significant relationship between collagen synthesis and component orientation during the manufacturing process.

In contrast, the processes characterized by the values for *MMP1* and *TIMP1* showed no significant influences due to the orientation during the manufacturing process.

With increasing roughness (Sa), the *MMP1/TIMP1* ratio decreased. The values for the SLM manufactured constructs were lower than those for the EBM manufactured processes. A weak linear relationship was found between the *MMP1/TIMP1* values and roughness as a result of the different build orientations. EBM constructs showed a higher correlation than the SLM constructs.

## 4. Discussion

We evaluated differently oriented component samples in terms of their surface roughness and their ingrowth behavior of osteoblastic cells by means of different cellbiological test methods. The main focus was on the comparative evaluation of the EBM and SLM test specimens as well as the evaluation of gene expression in comparison of the different orientations. Our particular interest was to generate additively manufactured titanium components to allow clear conclusions to be drawn about the surface properties and to provide a good possibility of cell seeding and subsequent cell biological assessment. In addition, the components provided the possibility to consider manufacturing-related surface properties in relation to the process, orientation in the installation space and orientation to the energy source (laser beam or electron beam).

The surfaces represented in Figure 2 show optically visible and quantitatively measurable differences. While the SLM and EBM manufactured components showed similar surfaces, the EBM manufactured components in the 45° and 90° orientations presented a coarser arrangement of the superficially present particles, as clearly seen. These visually recognizable differences can be demonstrated quantitatively by surface roughness measurements.

The roughness values (Sa) received for the manufactured Ti6Al4V specimens coincided with those from other investigations. The effects that influence surface roughness have already been described [22,33,36,37,38,39]. In particular, the energy flow into the powder bed and the orientation angle of the component play major roles. In addition to the orientation during the construction process, the construction parameters and the properties of the powder also influence the surface roughness. Our results confirm that the roughness depends on the orientation during the manufacturing process.

The assessment of the metabolic activities showed a significant correlation between specimen orientations and times of cell cultivation. Although differences in cellular metabolism based on surface orientation have been shown, our results indicate that, similar to other studies, the additive manufacturing processes by EBM and SLM specimens result in high biocompatibility [2,17,19,25] which was also confirmed by the live-dead staining.

In addition to roughness, the topography of the surface also influences the cellular activities, specifically the arrangement of particles on the surface (group of hills) as well as the laser-induced structure on the surface (lines representing the course of the laser). The observation of surface conditions and metabolic activities showed that small differences in surface structure effect the cell activity negatively. The SLM parts showed a decreasing level of cell activity from 0° to 45° to 90°. From the false color images in Figure 2, it is obvious that clear height differences (particle groups and line-shaped laser tracks) are associated with an increased activity level. A decrease in height difference also leads to a decrease in activity. This trend was confirmed in the EBM parts. High activity levels are associated with obvious height differences. A decrease in activities is also attributable to decreased structural differences. The EBM parts (45° oriented) show strong cell growth and, once again, the surface is characterized by large differences in the surface structure. Here, in addition to the accumulation of individual powder particles, areas that are rather flat and uniform can be seen.

In this work, interesting correlations were determined between roughness and the induction of bone formation/degradation processes. With regard to optimal osseointegration of such implant structures, a coordinated remodeling of surrounding bone tissue is required, and this process is controlled by balanced activity of MMPs and TIMPs [40]. Therefore, the induction of pro-osteogenic differentiation and the formation marker, *Col1A1*, as well as the matrix degrading protease, *MMP1*, and its natural inhibitor, *TIMP1*, was determined due to their involvement in bone matrix composition and turnover.

As shown in Figure 6, the bone formation process increases with increasing Sa. The average Sa and determined functional relationship demonstrate the induction of bone building processes. Larger roughness leads to stronger induction rates. This relationship applies equally to the SLM and EBM manufactured components. The determined functional relationships are proof of the supportive effect of rough surfaces on the bone formation process. The differences in the validity of such a relationship for both manufacturing variants were clearly pronounced after 7 days. The determined correlation coefficients supported these results. The induction of bone building process levels in the manufacturing variants was similar. The *Col1A1* values for the components of SLM (45° and 90°) corresponded to the *Col1A1* expression levels on the EBM parts (90°). In addition, there was a more pronounced increase in collagen 1 gene expression with human osteoblasts after 7 days than after 3 days at higher roughness.

This was also confirmed by the higher gene expression levels on the SLM components. Ponader et al. [20] assessed the suitability of different Ti6Al4V surfaces produced by the electron beam melting process. They found that on porous surfaces with higher Ra values, cell proliferation was reduced significantly [20]. In our present study, we observed the opposite effect. With regard to the processes, it can be concluded that the relationship also follows a temporal effect. With increasing time, the bone formation processes stabilize and follow a describable linear function.

Regarding the induction of bone degradation processes which are characterized by enhanced MMP1 and a concomitant reduction of TIMP1, it was shown that the orientation in the manufacturing process did not significantly influence the mRNA levels of both MMP1 and *TIMP 1*. It can be concluded that the manufacturing surface orientation could affect bone forming processes. However, we have to point out that we only determined gene expression rates. To confirm our conclusion, data of active protein levels need to be obtained in further analyses.

Consideration of the *MMP1/TIMP1* ratio provides an excellent way for determining the extent to which gene expression takes place. It is known that ratios less than 1 are indicators of events that are primarily attributable to building processes, whereas ratios higher than 1 indicate bone resorption events. On all investigated specimens, bone cells showed basically identical behavior in terms of gene expression rates. However, the functional correlation proved that the *MMP1/TIMP1* ratio decreased with increasing roughness. This indicates that surface roughness could directly influence expression profiles of important mediators. The EBM components also showed a weak functional relationship (for the MMP1/TIMP1 ratio and the roughness Sa) after 7 days. This correlation did not exist for the SLM components.

Overall, it is clear that the functional relationships are largely determined by roughness, which differs significantly. In addition, the characteristic values showed that the different manufacturing processes cause orientation dependent changes in roughness.

With regard to practical applications and manufacture of components for medical applications, slight advantages were shown for the SLM components since the behavior of these components for 45° and 90° was almost identical. The determined Col1A1 expression rates showed similar characteristic values for 45° and 90°. Since the production of complex components always represents an accumulation of differently oriented components, there were slight advantages in the production of components by means of SLM processes, as the 45° and 90° results hardly deviated from each other. The determined MMP1/TIMP1 ratios support the finding that the induction of beneficial bone formation processes prevails on the manufactured components.

The knowledge gained is important for use of endoprosthetic implants, which have different surface orientations. It offers design engineers the opportunity to customize the design of implants. However, collaboration with implant manufacturers is required in order to coordinate the design and manufacturing conditions (alignment and process selection). This offers new opportunities as well as challenges.

## 5. Conclusions

Test specimens made of Ti6Al4V were fabricated using the SLM and EBM processes. Their features were studied experimentally for three various build orientations. To investigate the influence of surface roughness on bone remodeling activities, the bone formation marker, *Col1A1*, as well as the relationship between the bone degradation marker, *MMP1*, to its natural inhibitor, *TIMP1*, were determined.

The following specific conclusions can be drawn:

- The roughness values (Sa) received for the manufactured Ti6Al4V specimens coincided with those from other investigations. The SLM parts showed significant lower Sa values.
- The orientations of the parts influenced the roughness and bone formation processes. Elevated cell viability was determined with increasing roughness. SLM manufactured Ti6Al4V parts induced higher formation processes at a concomitant reduction of the *MMP1* to *TIMP1* ratio. This was apparent after 7 days.
- In addition to roughness, the topography of the surface also influences the cellular activities.

In our in-vitro study, we could determine the influence of orientation on the biological properties of additively manufactured specimens. This knowledge offers a possibility for advanced design of titanium implants with respect to improved bone ingrowth. Developed know how can serve as guidance for the construction and subsequent placement or orientation during manufacturing of implant components to be used as bone substitutes.

## Figures and Tables

**Table 1:** Results from roughness measurements (Ra)—lengthwise and transverse—as well as the average arithmetic height (Sa) of the surface (*n* = 5).

Orientation	Ra–Lengthwise (μm)		Ra–Transverse (μm)		Sa (μm)	
	SLM	EBM	SLM	EBM	SLM	EBM
**0°**	1.33 ± 0.26	1.32 ± 0.18	2.63 ± 0.50	2.32 ± 0.21	7.12 ± 1.70	4.58 ± 1.02
**45°**	8.02 ± 1.89	9.37 ± 2.92	9.65 ± 2.29	11.56 ± 2.60	15.67 ± 2.62	30.77 ± 2.62
**90°**	9.83 ± 0.99	14.07 ± 2.10	10.63 ± 1.40	14.79 ± 1.65	14.62 ± 1.01	46.78 ± 4.44

**Table 2:** Gene expression analyses of bone formation (*Col1A1*) and degradation (*MMP1*, *TIMP1*) marker. Human osteoblasts were seeded on top of the different test specimens. After 3 and 7 days, RNA was isolated and reverse transcribed to determine mRNA levels of *Col1A1*, *MMP1* and *TIMP1* by qRT-PCR. Results are shown as mean values ± standard deviations (*n* = 3). Statistically significant results are marked with * for *p* < 0.05 and ** for *p* < 0.01 (repeated measures two-way ANOVA test with Bonferroni’s multiple comparison).

Orientation	Days	*Col1A1* Expression (2^−ΔCt^)		*MMP1* Expression (2^−ΔCt^)		*TIMP1* Expression (2^−ΔCt^)	
		SLM	EBM	SLM	EBM	SLM	EBM
**0°**	3	23,309 ± 8758	18,414 ± 7044	4.2 ± 2.0	4.2 ± 3.8	170 ± 22	155 ± 15
	7	16,830 ± 5068 *	11,642 ± 3574 **	10.2 ± 4.8	13.9 ± 8.6	201 ± 104	190 ± 15
**45°**	3	39,935 ± 24,747	29,252 ± 12,345	2.3 ± 2.6	2.6 ± 2.2	222 ± 72	150 ± 57
	7	43,482 ± 8016 *	24,553 ± 13,667	11.4 ± 9.8	8.6 ± 6.0	241 ± 40	159 ± 14
**90°**	3	35,449 ± 16,253	33,780 ± 7570	4.2 ± 3.8	1.9 ± 2.2	200 ± 33	176 ± 59
	7	37,029 ± 12,075	40,308 ± 8204 **	11.5 ± 8.4	6.8 ± 3.8	238 ± 28	251 ± 131
